# Breast cancer screening: evidence of benefit depends on the method used

**DOI:** 10.1186/1741-7015-10-163

**Published:** 2012-12-12

**Authors:** Philippe Autier, Mathieu Boniol

**Affiliations:** 1 International Prevention Research Institute, 95 Cours Lafayette, F-69006 Lyon, France

**Keywords:** breast cancer, case-control, effectiveness, epidemiology, incidence, mortality, screening

## Abstract

In this article, we discuss the most common epidemiological methods used for evaluating the ability of mammography screening to decrease the risk of breast cancer death in general populations (effectiveness). Case-control studies usually find substantial effectiveness. However when breast cancer mortality decreases for reasons unrelated to screening, the case-control design may attribute to screening mortality reductions due to other causes. Studies based on incidence-based mortality have obtained contrasted results compatible with modest to considerable effectiveness, probably because of differences in study design and statistical analysis. In areas where screening has been widespread for a long time, the incidence of advanced breast cancer should be decreasing, which in turn would translate into reduced mortality. However, no or modest declines in the incidence of advanced breast cancer has been observed in these areas. Breast cancer mortality should decrease more rapidly in areas with early introduction of screening than in areas with late introduction of screening. Nonetheless, no difference in breast mortality trends has been observed between areas with early or late screening start. When effectiveness is assessed using incidence-based mortality studies, or the monitoring of advanced cancer incidence, or trends in mortality, the ecological bias is an inherent limitation that is not easy to control. Minimization of this bias requires data over long periods of time, careful selection of populations being compared and availability of data on major confounding factors. If case-control studies seem apparently more adequate for evaluating screening effectiveness, this design has its own limitations and results must be viewed with caution.

See related Opinion article: http://www.biomedcentral.com/1741-7015/10/106 and Commentary http://www.biomedcentral.com/1741-7015/10/164

## Background

Four randomized trials conducted in Sweden from 1977 to 1993 obtained results suggesting 20% to 30% reductions in breast cancer mortality associated with the regular participation of women 40 to 74 years of age in mammography screening [1-4]. These trials encouraged the implementation of mammography screening services and, in 2012, screening programs have been in place for 20 years or more in several high income countries.

Breast screening mobilizes considerable resources and can generate harm due to false positive examinations, overdiagnosis and overtreatment, the amount of which depends on factors such as screening intensity, screening ages, radiologist experience and the legal environment (for example, defensive medicine in the US) [5-9]. An essential question is thus the effectiveness of screening activities, that is, are mortality reductions observed in the ideal conditions of randomized trials (efficacy) also found in the real world conditions of general population mass screening (effectiveness)?

In their paper, Puliti and Zappa [10] posit that case-control studies and studies based on incidence-based mortality (IBM) constitute valid, unbiased evaluations of screening effectiveness. By contrast, studies based on mortality statistics have limitations that explain why their findings are different from those of case-control and IBM studies. As a result of these considerations, the paper proposes to institute the case-control design as the standard method for evaluating breast screening effectiveness.

In this opinion article, we briefly review the main methods that have been used for evaluating the effectiveness of breast screening and discuss their strengths and weaknesses. Contrary to the opinion of Puliti and Zappa, we suggest that the case-control study design should not be used as the principal method to evaluate the effectiveness of screening programs.

### The case-control design

The case-control design compares the exposure to screening of women who died from breast cancer (the cases) to that of women alive and still at risk of dying from breast cancer at the time of death of the case (the controls). This comparison is deemed to inform on the ability of regular participation in screening to reduce the risk of dying from breast cancer. This design is appealing because of its low cost and swift execution [11].

The limitations of case-control designs for the evaluation of screening effectiveness have been described [12]. However, case-control studies done after 2000 were performed in populations where organized screening programs are established. These programs run databases collecting all of the screening information of women living in a country, including dates of invitation, participation, screening result and follow-up that can be matched with population-based cancer and cause of death registries. Selecting and matching cases and controls from such databases were deemed to guarantee equal opportunity of exposure to screening of both cases and of controls and to allow clear distinction between screening and diagnostic mammography [13].

Despite these improvements, a major problem that arises if the case-control study is conducted within the context of a population screening program is that women who do not participate in screening generally have a poorer outcome [14-17] for reasons that are not related to screening, thus inducing an observed lesser exposure to screening among the cases [18]. A growing number of data documents that, compared with women participating in screening mammography, non-participating women have characteristics associated with a higher risk of dying from breast cancer, such as higher rates of obesity and lower compliance to treatments, and the influence of these characteristics on the risk of breast cancer death exists in the absence of screening [19-21]. So, although a number of non-participants die from breast cancer for reasons unrelated to screening, results of case-control studies may suggest that these deaths are due to not having been screened. This bias in results has been termed the 'self-selection bias'. As the International Agency for Research on Cancer (IARC) Handbook on Breast Cancer Screening concluded, 'Observational studies of screening, such as cohort and case-control studies, may give biased measures of effect because of self-selection of women for screening. There are no certain ways of eliminating the bias' [22].

In 2002, Duffy *et al. *proposed a method to correct self-selection [18]. This method is based on computation of a quantity 'Dr' that is the ratio of the breast cancer mortality rate in non-participating women at the end of a screening period to the rate in women not invited to screening; that is, the rate in the control group of randomized trials on breast screening or the rate in an area before screening introduction. The quantity Dr is likely to vary among populations where screening efficiency is evaluated, because proportions of non-participants are variable. Dr is expected to be closer to zero when participation is low, because non-participants would be less atypical of the general population [18].

Table 1 summarizes the effects of correcting for self-selection in a population where breast screening is introduced. As said before, women not likely to participate in screening have a higher risk of breast cancer death that is independent of the existence of screening. Thus, before screening starts, the risk of breast cancer death of women likely to participate in screening is known to be 30% to 60% lower than that of women unlikely to participate [16-18]. For our example, we opted for a relative risk of breast cancer death of 0.60, meaning that before screening starts, the risk of death in participants was 40% lower than that in non-participants. The scenario in Panel A assumes no effect of screening on breast cancer mortality. A case-control study after a period of screening will find a crude risk of death of 0.61 in participants versus non-participants, but the correction for self-selection will yield an adjusted relative risk of 1.00, in agreement with no screening effectiveness. In Panel B, a 20% mortality reduction is obtained thanks to screening, and the correction leads to the right estimation of screening effectiveness. Panels C and D are similar to Panels A and B, but during the screening period, a 25% reduction change in mortality occurs that is unrelated to screening (for example, because of improved treatment). The Dr quantities are smaller because improved treatments have reduced the risk of breast cancer death in non-participants. As a consequence, the corrected relative risk in Panel C suggests that participation in screening is associated with a 44% (that is, 100 × (1.00-0.66)) reduction in breast cancer mortality, when in reality screening had no impact. The relative risk in Panel D is compatible with true screening effectiveness but the estimated mortality reduction of 52% (that is, 100 × (1.00-0.48)) is much larger than the 20% reduction actually due to screening.

**Table 1:** Relative risk of breast cancer death before screening start in women likely and not likely to participate in screening, and relative risk of breast cancer death after a period of screening with 75% participation.

		Before screening	After years of screening			**Correction according to Duffy *et al. *(2002) **[**18**]			
		
		BC death rate^a^	Change in mortality due to screening	Change in mortality unrelated to screening	BC death rate^a^	Dr	A = p × RR × Dr	B = 1-[(1-p) × Dr]	Corrected RR (= A/B)
**Panel A**	Participants	86	0%	0%	86				
	Non-participants	142	0%	0%	142				
	All women^b^	100	0%	0%	100				
	**RR breast cancer death, participants versus non-participants**	**0.61**			**0.61**	1.42	0.65	0.65	**1.00**
**Panel B**	Participants	86	-20%	0%	69				
	Non-participants	142	0%	0%	142				
	All women^b^	100	-15%	0%	85				
	**RR breast cancer death, participants versus non-participants**	**0.61**			**0.48**	1.42	0.52	0.65	**0.80**
**Panel C**	Participants	86	0%	-25%	65				
	Non-participants	142	0%	-25%	107				
	All women^b^	100	0%	-25%	75				
	**RR breast cancer death, participants versus non-participants**	**0.61**			**0.61**	1.07	0.48	0.73	**0.66**
**Panel D**	Participants	86	-20%	-25%	47				
	Non-participants	142	0%	-25%	107				
	All women^b^	100	-15%	-25%	60				
	**RR breast cancer death, participants versus non-participants**	**0.61**			**0.44**	1.07	0.35	0.73	**0.48**

BC: Breast cancer; Dr: rate in non-participants after screening divided by the rate in all women before screening; p: participation rate of 75%; RR: relative risk. ^a^Per 100,000 women-year. ^b^(rate in participants × 0.75) + (rate in non-participants × 0.25).

In areas where all women are regularly invited for screening, if breast cancer mortality changes for reasons other than screening, observational studies can hardly avoid the type of bias illustrated in Table 1. One could rely on breast cancer mortality rates in women of not (yet) invited women. However, breast cancer survival varies from region to region [23], and therefore, it is uncertain whether rates observed in uninvited women are valid for computing the quantity Dr. To be reliable, the quantity Dr needs to be assessed for each study, and should take into account changes in mortality due to factors other than screening in both unscreened and in uninvited women.

### Incidence-based mortality studies

Incidence based mortality (IBM) studies compare breast cancer mortality in patients with breast cancer diagnosed during similar periods before (pre-screening period) and after (screening period) screening introduction. Both breast cancer diagnosis and death must occur during the pre-screening or during the screening period. The advantage of this method is that it uses the 'refined mortality' that is obtained by excluding breast cancer deaths due to cancers diagnosed before start of the pre-screening period or before start of the screening period. IBM studies are challenging as they require first the possibility to link cancer registries to cause of death registries and second, to assemble the exact data on women who had breast cancer and died from it during the pre-screening and during the screening period.

According to the IARC Handbook on Breast Cancer Screening [22], 'Refined mortality should be estimated for screened and unscreened population to ensure comparability. Furthermore, cancer registration with data on treatment is likely to be the only means for differentiating the confounding effect of changes in treatment from the effect of screening.' Data on treatment is essential because variations in breast cancer mortality changes between regions may be due to differences in patient management rather than to screening, and a number of studies have documented substantial variability in patient management across regions, including in Sweden [23-26].

IBM studies on breast cancer screening [27-34] are summarized in Table 2. No study had data on patient management in pre-screening and in screening periods and thus no study could control for variations in patient management between areas. In all IBM studies, individual exposure to screening was generally known only for women diagnosed with breast cancer, but not for other women. Therefore, denominators (that is, person-years of exposure or non-exposure to screening) were estimated at population level, and not at individual level. In this respect, IBM studies needs to be considered as ecological studies.

**Table 2:** Summary of main incidence-based mortality studies on breast cancer screening.

Study	Country	Areas in study	Age of screened women	Data on treatments in screening and non-screening areas	Comparison areas where no screening existed during the entire screening period	Historical comparison areas^a^	Effect of screening on the risk of BC death	Adjustment of results for:		
								
								Self-selection	Incidence	Lead time
Tabár *et al. *2003 [27]	Sweden	Dalarna and Kopparberg counties	40 to 69	No	No	No	-45%	Yes	Yes	No
Olsen *et al. *2005 [28]	Denmark	Copenhagen where screening took place compared to the rest of the country	50 to 69	No	Yes	Yes	-25%	No	No	No
SOSSEG 2006 [29]	Sweden	About half of Swedish counties	40(50) to 69(74)	Yes, but only in non-participants	No	No	-27%	Yes	Yes	No
Jonsson 2007 [30]	Sweden	Four Swedish counties	40 to 74	No	Yes	No	-26%	Yes	No	Yes
Anttila *et al. *2008 [31]	Finland	All the country	50 to 69	No	No	No	-11%	No	No	No
Hellquist *et al. *2010 [32]	Sweden	All Swedish counties	40 to 49	No	Yes	No	-26%	Yes	No	Yes
Kalager *et al. *2010 [33]	Norway	Four counties with pilot program plus comparison counties	50 to 69	No	Yes	Yes	-10% (NS)	No	No	No
Olsen *et al. *2012 [34]	Norway	Four counties with pilot program plus comparison counties	50 to 69	No	Yes	Yes	-7 (NS)	No	No	No

BC: breast cancer; NS: statistically non-significant. ^a^Data on breast cancer death rates in historical comparison groups, that is, in screening areas and in comparison areas before screening started in screening areas.

An IBM study in Finland based on data from the Finish Cancer Registry found an 11% mortality reduction [31]. All four Swedish IBM studies obtained results indicating considerable screening effectiveness [27,29,30,32]. However, in Sweden, breast cancer mortality has steadily decreased by 0.9% per year since 1972, well before screening started [35,36]. Because they had no comparison areas where no screening existed during the entire study period, two IBM studies done in Sweden [27,29] could not realize how these secular trends influenced their results.

Swedish IBM studies corrected their results in various ways (Table 2). Two studies corrected changes in breast cancer mortality for changes in breast cancer incidence [27,29]. This correction is based on the assumption that if screening had not existed, trends in breast cancer mortality would have paralleled incidence trends. This correction is not justified because first, in the absence of screening, mortality and incidence trends are not correlated. Second, mammography screening itself is the main cause of increasing breast cancer incidence. The correction for lead time is also questionable, as lead time is known to affect survival statistics but not mortality [37].

Three IBM studies, one in Denmark [28] and two in Norway [33,34], included comparison areas as well as historical areas (that is, the screening and comparison areas before screening start) where women were not invited to screening (Table 2). The two studies in Norway were based on the same screening areas but used different comparison areas. The study in Denmark found a statistically significant 25% mortality reduction associated with screening, while the two studies in Norway found 10% and 7% reductions that were not statistically significant.

Despite comparable design, IBM studies in Denmark and in Norway differed in several ways (Tables 2 and 3). In Denmark, screening was mainly performed in Copenhagen, the capital city. The data source for breast cancer cases was the Copenhagen mammography screening register, whereas for the rest of the country, a comparison group was constructed from the central population register that was linked to the Danish Cancer Registry. In Norway, screening was initiated in four counties; in both studies, the data source for breast cancer cases was the Norwegian Cancer Registry. In Denmark, in the pre-screening period, the mortality rate of Copenhagen women was 33% higher (that is, 69 compared with 52) than among women living elsewhere in Denmark (Table 3). Also, in comparison areas, from the pre-screening to the screening period, no decline in mortality rates occurred. Such contrast between the screened and comparison areas did not exist in the Norwegian studies. It is probable that the results of the Danish study were owing to differences in ways by which breast cancer cases were assembled in screened and in comparison areas.

**Table 3:** Incidence-based mortality studies for the evaluation of mammography screening effectiveness in Denmark and in Norway.

	Years included in study	Year of screening introduction in screening areas	Age groups	BC mortality rate in the pre-screening period (historical control)	BC mortality rate in the screening period	Ratio of BC mortality rates, screening versus pre-screening period^a^	Ratio of BC mortality rates, screening versus non-screening areas^a^
***Screening areas***				No screening	Screening		
Denmark, Olsen *et al. *2005 [28]	1981 to 2001	1991	50 to 79	69	52	0.75	0.74
Norway, Kalager *et al. *2010 [33]	1986 to 2005	1996	50 to 84	33	24	0.73	0.9
Norway, Olsen *et al. *2012 [34]	1990 to 2008	1996	50 to 84	35	27	0.77	0.91
***Comparison (non-screening) areas***				No screening	No screening		
Denmark, Olsen *et al. *2005 [28]	1981 to 2001	-	50 to 79	52	53	1.02	reference
Norway, Kalager *et al. *2010 [33]	1986 to 2005	-	50 to 84	31	25	0.81	reference
Norway, Olsen *et al. *2012 [34]	1990 to 2008	-	50 to 84	39	33	0.85	reference

Figures in the table are breast cancer mortality rates per 100,000 women ages 50 to 79 in Denmark and 50 to 84 in Norway. ^a^Rate ratios may slightly differ from those of the original publications reported in Table 2 due to rounding and absence of statistical modelling. BC: breast cancer; NR: not reported.

A final point is that IBM studies could underestimate mortality reductions if substantial mammography screening is already present in comparison areas. Detailed information on the provision of mammography examinations in Sweden and Norway indicated that mammography screening outside the national breast screening programs was uncommon [38-40].

### Change in the incidence of advanced breast cancer

Clinical data show a strong correlation between the size of breast cancer and the likelihood of metastases in axillary lymph nodes or in distant organs. Breast cancer screening is based on the principle that detection of a cancer when still small and not symptomatic prevents progression to advanced disease associated with positive lymph nodes or distant metastases. The consequence of early detection would be a reduction of the risk of death from breast cancer.

Monitoring the incidence of advanced cancer thus corresponds to the way screening works. In addition, the incidence of advanced breast cancer is not influenced by subsequent treatments. Therefore, in populations where breast screening has been widespread for a long period of time (say, seven years or more), a reduction of advanced cancer incidence should reflect the impact of screening activities alone. Longstanding broad consensus exists for considering a decrease in advanced breast cancer incidence as the best early indicator of the impact of screening [1,22,41-43]. This consensus was in agreement with cancer registry data showing marked decreases in the incidence of advanced cervical and colorectal cancers over the last decades [44,45], which illustrated the contribution of screening to the reductions in mortality from these two cancers. Of note, decreases in the incidence of advanced breast cancer after screening introduction should not be confused with decreases in proportions of advanced breast cancer [22]. The latter does not necessarily reflect the influence of screening because screening will increase numbers of early cancers detected, due to lead time (advance in diagnosis) and to length time (overdiagnosis). Increases in the number of early cancer arithmetically lead to increases in proportions of early cancer that in turn lead to decreases in proportions of advanced cancer. Therefore, published data show that proportions of advanced cancer usually diminishes in years following breast screening introduction while concomitant reductions in the incidence rate of advanced cancer may be modest or absent.

The IARC meeting of 2002 devoted a section on trends in advanced breast cancer incidence [22]. However, at that time, few cancer registries had collected adequate data over a too short period after screening introduction.

From 2006 onwards, with accumulating years of screening activities in populations where good quality cancer registries exist, larger amounts of data on advanced breast cancer incidence were available. A systematic review showed that, in areas in Europe, North America and Australia where screening has been widespread for a long time, no or small decreases in the incidence of advanced and very advanced breast cancer was observed [46]. An analysis of breast cancer incidence in the US reached the same conclusion [47]. A team of radiologists performed an in-depth analysis of screen-detected, interval and all breast cancers diagnosed from 1989 to 2007 in the south-east region of the Netherlands and found no decline in the incidence of advanced breast cancer [48]. In the UK, cancer registry data for Scotland, Northern Ireland and the West Midlands showed no decline in the incidence of advanced breast cancer after the introduction of screening in 1989 [46,49]. In Norway, the study that found a modest non-significant reduction in the risk of breast cancer death in screened versus non-screened populations [33] also found no difference in rates of advanced breast cancer in these populations [39].

A question is whether the incidence of advanced breast cancer would have increased in the absence of screening. Few data indicate that in areas where screening has been widespread for some time, the answer is likely to be negative. In the Netherlands, where screening before 50 and above 69 years of age was rare, there were no time changes in the incidence of advanced breast cancer in women younger than 50 and older than 69 years of age [42,50]. In Victoria (Australia), the trends in the incidence of advanced breast cancer in non-attending women were similar to that observed among screened women [51].

### Changes in breast cancer mortality rates in areas with large difference in the timing of screening introduction

If breast screening was capable of reducing breast cancer mortality by 20% to 30% after seven to ten years, reductions should be quicker and more apparent in countries with early screening implementation whereas delayed and smaller reductions should be observed in countries with late implementation.

The ecological design may be useful for comparative effectiveness research, that is, comparison of disease-specific trends in countries with similar quality of health systems and access to treatment, but with different prevention policies. This design has been used when randomized trials were unfeasible, for example the banning of smoking in public places in 2006 in Scotland was followed by a one-year 17% reduction in hospital admissions for acute myocardial events [52]. By contrast, in England where such a ban did not yet exist, the hospital admission during that one-year period decreased by 4% [52]. In this respect, the IARC Handbook on Breast Cancer Screening stated that, 'Routine screening programmes can be evaluated most readily by time trends and differential mortality from the disease for which screening is being performed. Probably the best known is screening for cervical cancer. The substantial differences among the Nordic countries in the extent of organized screening were closely matched by the mortality rates from cervical cancer (Läärä et al., 1987)' [22].

Another study mimicked the Nordic study on cervical cancer screening [53] by selecting three pairs of European countries (the Netherlands and Belgium; Northern Ireland and Ireland; Sweden and Norway) with similar prevalence of risk factors for breast cancer death, access to treatment and expenditures for health, but where by year 1993, nationwide screening was in place in the first country of each pair, but implemented 10 to 12 years later in the second country [54]. In each country pair, breast cancer mortality rates in 1987 to 1989 were comparable and from 1989 to 2007, equivalent declines in breast cancer mortality occurred. These results agreed with the observation that breast cancer mortality reductions in high income countries are unrelated to the temporal introduction of screening mammography [35,55].

Perhaps a longer period would be needed to observe the benefits of screening. However, in the Netherlands and the UK, changes in mortality observed six to ten years after screening introduction were attributed to screening [56,57]. In this respect, the 18-year-long period used in country pairs [54] was long enough to observe the impact of screening on mortality. Another limitation is the contamination of mortality data during the screening era with deaths due to breast cancer diagnosed before screening start. In 1993, the eight-year survival of deadly (stage III) breast cancer was about 30% [58]. The study on country pairs [54] extended over 15 to 19 years after screening start. Hence most fatal breast cancers diagnosed before the screening era weighted little in the breast cancer mortality burden after 2000.

Two other studies on long-term breast cancer mortality trends according to timing of screening introduction were conducted in Danish regions and in Swedish counties [36,59]. During the 15 years following screening start, no change in the difference in mortality trends was noticeable between the screened and unscreened Danish regions. In Swedish counties, mortality rates continued to follow the monotonous downward trends that started in 1972, without sign that screening may have influenced these trends.

## Conclusions

Case-control studies are relatively easy and quick to perform. However, when cancer mortality is decreasing for reasons unrelated to screening, this design may not be able to control the self-selection bias, therefore deaths prevented through other interventions may be attributed to screening. In this respect, contrary to the suggestion of Puliti and Zappa [10], a case-control study should not be considered as the standard method for the evaluation of breast screening effectiveness.

IBM studies focus on deaths from breast cancer occurring during periods of the same duration before and after screening introduction. But results from IBM studies seem to vary according to the design adopted and adjustments performed during the statistical analysis. Also, results may be biased if secular trends in mortality, improved treatment and geographical variations in patient management are not considered.

The method based on incidence trends of advanced breast cancer corresponds to the primary goal of screening that consists in preventing advanced cancer. Its main limitation is the paucity of population-based cancer registries that have collected data on breast cancer characteristics over long periods of time. Monitoring incidence trends in women living in areas with screening but not invited to screening is desirable for verifying that the lack of decreasing trends in advanced cancer is not due to the increasing prevalence of factors associated with greater risk of advanced breast cancer.

A comparison of mortality trends between screened and unscreened areas takes into consideration factors influencing mortality in the areas being compared, but several methodological requirements need to be met for minimizing ecological biases, such as a period of observation that is long enough, availability of data on participation in screening during the observation period, and availability of data on major confounding factors like access to treatment.

Studies based on time trends of advanced disease incidence and cancer-specific mortality, with use of designs enabling comparisons between periods and geographical areas, have documented that screening for cervical and colorectal cancer contributed to decreasing mortality from these two cancers [44,45,53]. We thus conclude that, similarly, an evaluation of the effectiveness of breast screening should be based on monitoring of advanced cancer incidence, as well as on mortality trends in areas with contrasting screening policies where good information is available on non-screening factors involved in mortality, so that ecological biases can be minimized. Indeed, it remains to be explained why results from these two types of studies on breast screening effectiveness do not match the results of Swedish randomized trials.

## Competing interests

The authors declare that they have no competing interests.

## Authors' contributions

PA drafted the article, PA and MB summarized published data and formulated the statistical example, PA and MB discussed the content. Both authors approved the final version of the manuscript.

## Pre-publication history

The pre-publication history for this paper can be accessed here:

http://www.biomedcentral.com/1741-7015/10/163/prepub
